# The Pediatric Guideline Adherence and Outcomes (PEGASUS Argentina) program in severe traumatic brain injury: study protocol adaptations during the COVID-19 pandemic for a multisite implementation-effectiveness cluster randomized controlled trial

**DOI:** 10.1186/s13063-022-06938-x

**Published:** 2022-12-05

**Authors:** Julia Velonjara, Brianna Mills, Silvia Lujan, Gustavo Petroni, Michael J. Bell, Nahuel Guadagnoli, Charles Mock, James P. Hughes, Monica S. Vavilala, Ali Rowhani-Rahbar, for the PEGASUS Argentina Study Group, Mariela Alassia, Silvina Abalos, Natalia Gómez Arriola, Pablo Castellani, Sandra Chuchuy, Karina Cinquegrani, Carlos Dávila, Adriana Diettes, Gabriela López Cruz, Alejandro Mansur, Ivana Marinelli, Paula Medici, Silvia Oliveri, Matías Penazzi, Graciela Romero, Ariel Segado, Alejandra Depetris, Daniel Giordano, Linda Ng Boyle, Megan Moore, Bryan Weiner, Karen Segar, Jin Wang, Shyam J. Deshpande, Chelsea Hicks, Janessa Graves

**Affiliations:** 1 University of Washington Department of Anesthesiology & Pain Medicine, Seattle, WA USA; 2 Harborview Injury Prevention & Research Center, Harborview Medical Center, Box 359660, 325 Ninth Avenue, Seattle, WA 98104 USA; 3 University of Washington Department of Epidemiology, Seattle, WA USA; 4 Centro de Informática e Investigación Clínica (CIIC), Rosario, Argentina; 5 Pediatrics, Critical Care Medicine, Children’s National Hospital, Washington, DC USA; 6 University of Washington Department of Biostatistics, Seattle, WA USA

**Keywords:** Study design changes, Protocol, COVID-19 pandemic impact, TBI

## Abstract

**Background:**

The aim of this protocol is to describe the study protocol changes made and subsequently implemented to the Pediatric Guideline Adherence and Outcomes (PEGASUS) Argentina randomized controlled trial (RCT) for care of children with severe traumatic brain injuries (TBI) imposed by the COVID-19 pandemic. The PEGASUS study group met in spring 2020 to evaluate available literature review guidance and the study design change or pausing options due to the potential interruption of research.

**Methods:**

As a parallel cluster RCT, pediatric patients with severe TBIs are admitted to 8 control (usual care) and 8 intervention (PEGASUS program) hospitals in Argentina, Chile, and Paraguay. PEGASUS is an intervention that aims to increase guideline adherence and best practice care for improving patient outcomes using multi-level implementation science-based approaches. Strengths and weaknesses of proposed options were assessed and resulted in a decision to revert from a stepped wedge to a parallel cluster RCT but to not delay planned implementation.

**Discussion:**

The parallel cluster design was considered more robust and flexible to secular interruptions and acceptable and feasible to the local study sites in this situation. Due to the early stage of the study, the team had flexibility to redesign and implement a design more compatible with the conditions of the research landscape in 2020 while balancing analytical methods and power, logistical and implementation feasibility, and acceptability. As of fall 2022, the PEGASUS RCT has been active for nearly 2 years of implementation and data collection, scheduled to be completed in in fall 2023. The experience of navigating research during this period will influence decisions about future research design, strategies, and contingencies.

**Trial registration:**

Pediatric Guideline Adherence and Outcomes-Argentina. Registered with ClinicalTrials.gov Identifier NCT03896789 on April 1, 2019.

**Supplementary Information:**

The online version contains supplementary material available at 10.1186/s13063-022-06938-x.

## Administrative information

Note: the numbers in curly brackets in this protocol refer to SPIRIT checklist item numbers. The order of the items has been modified to group similar items (see http://www.equator-network.org/reporting-guidelines/spirit-2013-statement-defining-standard-protocol-items-for-clinical-trials/).

**Table:** 

Title {1}	The Pediatric Guideline Adherence and Outcomes (PEGASUS Argentina) program in severe traumatic brain injury: study protocol adaptations during the COVID-19 pandemic for a multisite implementation-effectiveness randomized controlled trial
Trial registration {2a and 2b}.	Pediatric Guideline Adherence and Outcomes-Argentina. Registered with ClinicalTrials.gov Identifier NCT03896789 on April 1,2019.
Protocol version {3}	V.3 June 2020
Funding {4}	Research is supported by the National Institute of Neurological Disorders and Stroke of the National Institutes of Health under award number R01NS106560 (PIs: Vavilala, MS and Bell, MJ). Primary award is to the University of Washington, subcontracts awarded to support CIIC, CNH, and WSU. Study hospitals receive funding through CIIC based on patient enrollment as well as subsidized travel.
Author details {5a}	JV, MSV-University of Washington Department of Anesthesiology & Pain Medicine, Seattle, WA, JV, BM, CM, MSV, ARR-Harborview Injury Prevention & Research Center, Seattle, WA, BM, ARR-University of Washington Department of Epidemiology, Seattle, WA, SL, GP, NG-Centro de Informática e Investigación Clínica (CIIC), Rosario, Argentina, MJB-Pediatrics, Critical Care Medicine, Children's National Hospital, Washington DC, USA., JPH-University of Washington Department of Biostatistics, Seattle, WA
Name and contact information for the trial sponsor {5b}	The University of Washington is the primary recipient and sponsor, and Carol Rhodes (osp@uw.edu) is the Authorized Official.
Role of sponsor {5c}	The sponsor and funder had no role in the design of this study and will not be involved in the implementation, analyses, interpretation of data, or writing of manuscripts for publication.

## Introduction

### Background and rationale {6a}

Globally, over three million children sustain a traumatic brain injury (TBI) each year, with an estimated 3–7% being classified as having a severe TBI (Glasgow Coma Scale (GCS) score ≤ 8). Long-term TBI-related disability and high health care costs are a significant burden to individuals and society, although data are limited outside of the USA and Europe [1, 2]. The Brain Trauma Foundation (BTF) Guidelines for Management of Pediatric Severe TBI represent the most comprehensive synthesis of evidence-based care for children with severe TBI [3], but they are not systematically implemented [4] and are also almost exclusively based on evidence from research in the USA and Europe.

Pediatric Guideline Adherence and Outcomes (PEGASUS) Argentina is a National Institutes of Health (NIH)-funded, multi-site randomized controlled trial (RCT) currently testing implementation of best practice care for children with severe TBI in South America [5]. The study is registered with ClinicalTrials.gov (Table 1). The single center PEGASUS program pilot at Harborview Medical Center (level 1 pediatric trauma center in Seattle, Washington) demonstrated improved adherence to key performance indicators of the BTF guidelines and patient outcomes, while not increasing in-hospital costs [6, 7]. While feasible, acceptable, and efficacious in the pilot study, the next step was to test PEGASUS intervention effectiveness more broadly. Focus on our partnership with experienced TBI researchers in Argentina at the Centro de Informática e Investigación Clínica (CIIC), and implementation at South American hospital sites will expand evidence of which guidelines are most important contributors to 3-month patient outcomes and examine generalizability of the PEGASUS program.

**Table 1:** ClinicalTrials.gov registration data

Data category	Information
NCT Number ^ICMJE^	NCT03896789
Other Study ID Numbers ^ICMJE^	STUDY00005629 1R01NS106560-01A1 ( U.S. NIH Grant/Contract)
Last Update Posted Date	December 10, 2021
First Submitted Date ^ICMJE^	January 7, 2019
First Posted Date ^ICMJE^	April 1, 2019
Primary Outcome Measures ^ICMJE^ (submitted: March 27, 2019)	TBI guideline adherence [Time Frame: ICU Stay, approximately up to 2 weeks ] The main outcome will be measured as the sum of indicators to which care was adhered by the number of relevant adherence indicators for a given patient during ICU stay.
Secondary Outcome Measures ^ICMJE^ (submitted: November 25, 2019)	• Clinical Pathway Adoption [ Time Frame: within 24 hours of patient admission ] • Discharge Survival [ Time Frame: At Hospital Discharge, approximately up to 5 weeks ] • GOSE-Peds. Pediatric Version of the Glasgow Outcome Scale-Extended [ Time Frame: 3 months post discharge ] • Mortality [ Time Frame: 3 months post discharge ] • DIBQ (Determinants of Implementation Behaviors Scale) [Time Frame: Baseline, and quarterly during year 1 after randomization; then once annually through study completion, approximately 3 years, for intervention sites.] • Value added processes assessed by Organizational Questionnaire for Participant Hospitals [Time Frame: Baseline and annually through study completion, approximately 3 years] • Changes in patient outcomes from time in - to time out- of the system based on manipulations of KPIs [Time Frame: during ICU care, approximately up to 2 weeks]
Brief Title ^ICMJE^	Pediatric Guideline Adherence and Outcomes- Argentina
Official Title ^ICMJE^	Implementation Fidelity and Benefits of the Critical Care Pediatric Guideline Adherence and Outcomes Program in Traumatic Brain Injury
Study Type ^ICMJE^	Interventional
Study Phase ^ICMJE^	Not Applicable
Study Design ^ICMJE^	Allocation: Randomized Intervention Model: Parallel Assignment Intervention Model Description: Parallel cluster RCT following a baseline data collection period when all sites are in the 'control/usual care state'. Masking: None (Open Label) Primary Purpose: Health Services Research
Condition ^ICMJE^	TBI (Traumatic Brain Injury)
Intervention ^ICMJE^	Other: PEGASUS Program for Care This is in essence a checklist of pediatric guidelines to follow for participants who meet inclusion criteria. Site staff will receive training in how to implement this pathway into their usual care.
Study Arms ^ICMJE^	• No Intervention: Baseline All sites will collect usual care data from 9 months to 21 months. • Experimental: PEGASUS Program (Intervention) This arm (half of the sites) will receive the PEGASUS program (intervention) from 21 months to 57 months. Intervention: Other: PEGASUS Program for Care • No Intervention: Usual Care (Control) This arm (half of the sites) will maintain usual care. They will receive the opportunity for the PEGASUS program training (intervention) at the end of study data collection (57 months) period.
Recruitment Status ^ICMJE^	Recruiting
Estimated Enrollment ^ICMJE^ (submitted: March 27, 2019)	540
Actual Study Start Date ^ICMJE^	September 1, 2019
Estimated Primary Completion Date	September 30, 2023 (Final data collection date for primary outcome measure)
Estimated Study Completion Date ^ICMJE^	December 31, 2023
Eligibility Criteria ^ICMJE^	Inclusion Criteria: • Mechanism or head CT consistent with TBI • < 18 years old • GCS (Glasgow Coma Scale) score ≤8 at any point during hospital admission Exclusion Criteria:- none
Listed Location Countries ^ICMJE^	Argentina, Chile, Paraguay
Contacts ^ICMJE^	Julia Velonjara, MPH-julialv@uw.edu Monica Vavilala, MD-vavilala@uw.edu
Responsible Party	Monica Vavilala, University of Washington
Funding Support ^ICMJE^	National Institute of Neurological Disorders and Stroke, National Institutes of Health
Study Sponsor ^ICMJE^	University of Washington
Collaborators ^ICMJE^	• Children's National Research Institute • Centro de Informática e Investigación Clínica (CIIC) • Washington State University • National Institute of Neurological Disorders and Stroke

### Objectives {7}

The specific aims are:

1. Determine the relationship between PEGASUS program implementation and TBI guideline adherence (aim 1a) and assess system, provider, patients, implementation, and guideline factors associated with TBI guideline adherence (aim 1b).
2. Create a value stream map (VSM) that readily identifies value added process of care associated with TBI guideline adherence.
3. Use computer simulation to develop and disseminate a real-world best practices blueprint for TBI guideline adherence. This is a necessary advance and a step towards implementing guideline based TBI care for children who suffer from TBIs.

### Trial design {8}

The purpose of this manuscript is to describe the precedence, considerations, decision-making process, and proposed options to the PEGASUS Argentina study design protocol in response to the COVID-19 pandemic, as well as examine the status of the implemented design 2 years later. We describe elements of the study that allowed for potential interruptions and design changes. We will use the CONSORT Extension to Cluster RCTs in reporting trial features and findings.

**Fig. 1:**
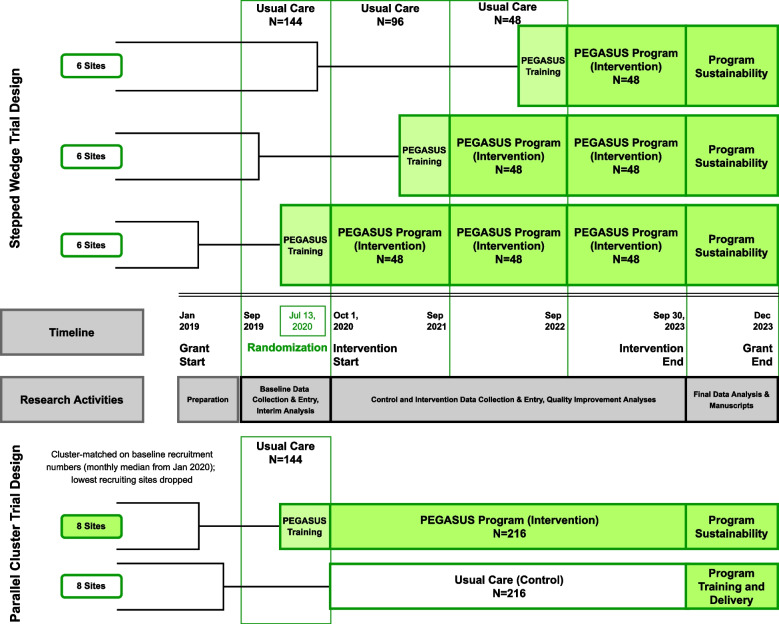
Comparison of PEGASUS stepped wedge and parallel cluster designs stepped wedge design implemented September 2019–May 2020. Parallel cluster design developed April–May 2020, implemented May 2020

The PEGASUS Argentina study is a pragmatic implementation-effectiveness cluster RCT design collecting data and implementing the intervention bundle over 3 years. The multi-level PEGASUS program targets improving guideline adherence to the best standards of care through quality improvement strategies. The study collects electronic health record and PEGASUS specific patient data in-hospital and at three months, as well as qualitative components engaging hospitals and providers on implementation and quality improvement. During initial in-person site visits and study planning, and based on feedback from Argentine site investigators, motivated hospital sites, and local institutional review boards (IRB), the study team developed a stepped wedge cluster study design with a goal to facilitate equitable site access to the PEGASUS program [8]. Additional sites were recruited in order to have a built-in buffer of sites with sufficient recruitment by the beginning of the intervention implementation. Despite added complexity in statistical analysis with the stepped wedge design, all sites would be assigned to the intervention over the course of the study thereby facilitating equity in the receipt of the program. The logistics of training additional sites would be evenly spread out over the study period. All sites launched baseline data collection in September 2019, with the first wave crossing over from usual care to the PEGASUS program intervention planned for October 2020, with subsequent annual waves in 2021 and 2022, and the completion of data collection scheduled for September 2023 (Fig. 1).

When the World Health Organization declared the COVID-19 pandemic in March 2020, PEGASUS Argentina had collected 6 months of pre-intervention data but had not yet randomized or trained sites for the first wave of the PEGASUS program intervention. The study group began to discuss research adaptations and contingency planning due to ongoing uncertainty about the impact and duration of the COVID-19 pandemic globally and the effects on the PEGASUS Argentina study specifically. After a few months of deliberation about the uncertain secular effects of the COVID-19 pandemic on stepped wedge design, and to mitigate the impact of secular effects of the pandemic relatively early stage of the study, the study group instead implemented a parallel cluster RCT study design (Fig. 1).

#### Literature review

As we anticipated the necessity of adapting our ongoing study, we conducted a PubMed literature review to assess any available information on situations that paused, stopped, or caused other design changes to in-progress RCTs from the past 10 years [9]. Key search terms included the following: discontinuation, early stopping, study pause, study completion, study design change, nonpublication, critical care, pediatrics, and RCT. Article titles and abstracts were screened for relevancy to our inquiry. If applicable, the full article was consulted, key points compiled, and citations reviewed for additional articles. At this stage (early April 2020), we also checked clinical trials records for recent status changes to recruiting studies [10].

Prior to 2020, some RCTs changed study plans or ended early for reasons particular to the context of a study [11]. Several reviews on discontinuation and non-publication of pediatric RCTs estimated that 15–40% of trials were ended early, most often citing reasons related to low patient recruitment or informed termination, either for early superiority or futility [11–16]. Few reviews specified ‘suspended’ studies separately, grouping them with those that had been ‘discontinued’ [12–14], and there were few identified cases of ‘suspended’ studies that were restarted and went on to be completed and published.

#### Study group-led changes

The study group, in consultation with a researcher with stepped wedge design expertise (JPH), met throughout March, April, and May 2020 to systematically compare potential study designs’ strengths and weaknesses related to analytical methods, logistical and implementation feasibility, acceptability, and other factors. We recognized that different stakeholders in the study may have had different preferences on the course of action, but our priority was to balance those while enabling our study to continue safely. To be transparent and open with the local site principal investigators (PIs) and IRBs, their assent for the resulting recommendation was included in the process. Due to the COVID-19 pandemic, meetings were held via Zoom to evaluate our final choice of RCT.

The PEGASUS Argentina study group relies on regular remote communication methods and was being updated by the Argentina-based co-investigators as the incidence of COVID-19 increased and lockdown measures were enacted [17]. PEGASUS Argentina site PIs and site coordinators were able to continue baseline data collection uninterrupted because it relied solely on medical charts. Eligible patients were admitted to the pediatric intensive care units (PICU) with severe TBI despite local pandemic situations. They could be recruited by the staff who were already present as part of their clinical roles and accustomed to this consent process over the past 6 months. The patient PEGASUS intervention is also delivered as part of clinical care. The site training and quality improvement components could be delivered remotely. However, the study group was concerned about pandemic fluctuations impacting the implementation of the PEGASUS program, limiting validity of analysis, and introducing further unexpected constraints and confounding. The sample size and power calculations for the study designs are summarized in Table 2.

**Table 2:** Comparison of assumptions and sample size calculation for power parallel cluster and stepped wedge designs

Design	Sites	Assumptions	Participants	Alpha	Power	ICC	Detectable difference
**Initial parallel RCT**	6	Adherence 58.6% No change in control group adherence	540 total	0.05	80%	0.01	15.5%
						0.05	24.6%
						0.10	31.1%
*Options 1 and 2:* **Stepped wedge**	12	Adherence 58.6% No change in control group adherence	432 total	0.05	80%	0.05	19.7%
						0.10	19.8%
						0.20	19.1%
*Option 3:* **Final parallel RCT**	16	Adherence 58.6% No change in control group adherence	576 total	0.05	80%	0.05	18.0%
						0.10	22.5%
						0.20	28.8%

*ICC* intraclass correlation coefficient, *RCT* randomized controlled trial

#### Proposed options

Three main options for RCT design choice were identified and examined: (1) continue with stepped wedge design as planned, (2) postpone intervention start but keep stepped wedge design, or (3) convert to a two-arm parallel cluster design randomized on baseline enrollment to balance future participants across arms. For each option, we factored in the effect of design change on sample size and power to ensure that our study analysis would still be able detect differences with the anticipated number of participants over the recruitment period.

#### Option 1

Continuing with the established stepped wedge design strengths included no added work for staff to adapt designs and study plans and aligned with site PIs’ initial desire to all receive the intervention. Weaknesses included possible study interruption if circumstances made it impossible to recruit participants or deliver the intervention, less resilience to reduced power if recruitment is much lower than previously estimated, and increased complexity of analysis and confounded results due to secular trends limiting sites’ ability to follow the pre-specified stepped wedge schedule. This situation could essentially revert the intervention from a “randomized” study to an “observational” study. The estimation for detectable percentage point differences in guideline adherence at alpha 0.05, 80% power, and intraclass correlation coefficients (ICC) ranging from 0.05 to 0.2 was between 19.1 and 19.7%.

#### Option 2

Maintaining the stepped wedge design but postponing the intervention start could have given more time to identify COVID-19 impacts to the study and lower demand and stress on sites during the pandemic. This option would have many of the same drawbacks as option 1. Additionally, the uncertainty of delay and unclear thresholds for when the situation would improve so implementation could begin would result in a shorter data collection period of therefore reducing recruitment and power, as well as potential loss of motivation for the entire study group. If the study were extended to offset the reduced data collection period, further expenses would be incurred. The same power calculations pertain as in option 1.

#### Option 3

Some strengths of a two-arm parallel cluster design would be the ability to maintain valid comparison between the PEGASUS intervention and control arms and a more robust, flexible design for secular interruptions like if the study needed to be paused. Temporal impacts of the pandemic would at least equally affect the two parallel arms, as opposed to the stepped wedge, where the time specific waves would have sites beginning intervention in multiple different contexts. This more traditional two-arm model would also maintain better statistical power if recruitment was less than anticipated. Option 3 weaknesses included the need for renewed site PI and local IRB buy-in for changes, some sites would not receive the intervention, and staff burden to prepare and implement changes to all intervention sites within a condensed time period than was previously planned. The estimation for detectable percentage point differences in guideline adherence at alpha 0.05, 80% power, and ICC ranging from 0.05 to 0.2 was between 18.0 and 28.8%. This two-arm parallel cluster design option was selected, implemented, and described in the following sections.

## Methods: participants, interventions, and outcomes

### Study setting {9}

The final study sites are sixteen PICUs at hospitals who regularly treat pediatric patients with severe TBI. Hospitals provided information on initial hospital characteristics and patient population during site selection. Fourteen hospitals are in cities in Argentina, one in Chile, and one in Paraguay [5].

### Eligibility criteria {10}

PICU staff screens patients admitting to the PICU for inclusion based on the following criteria: (1) age ≤ 18 years at admission, (2) mechanism of injury and head CT consistent with TBI, and (3) GCS ≤ 8 at any point during their stay. If eligible, the 24-h-on-call study coordinator is notified to begin a culturally appropriate consent process.

### Who will take informed consent? {26a}

Both the control sites and intervention sites approach eligible patients for consent. The attending physician introduces the family or other legal guardian to the study coordinator and makes sure the family knows the credentials and purpose. The study coordinator initiates a confidential conversation introducing research and describe all relevant aspects of the project, as well as answering questions and assessing understanding. The study coordinator assures the family that they are free to decline consent without consequences and that they can withdraw consent at any time. The family provides written consent and oral assent will be sought when a child > 7 years old regains consciousness (Additional file 1). Family members will be provided with contact information for the site PI and coordinator, CIIC, and the local ethical committee. Enrolled participants at the control sites consent to data collection from their medical records. Enrolled participants at the intervention sites consent to data collection and receive the intervention delivered by the trained PICU staff delivering their care.

### Additional consent provisions for collection and use of participant data and biological specimens {26b}

N/A. No biological specimens are being collected.

### Interventions

#### Explanation for the choice of comparators {6b}

Intervention hospital sites implement a program bundle which aims to increase adherence to BTF guidelines, while control hospital sites continue with their usual care for pediatric TBI.

#### Intervention description {11a}

The PEGASUS program intervention has multi-level components to increase guideline adherence. The patient level clinical care pathway is implemented for each eligible, consented patient at an intervention site for up to 7 days of their ICU stay. Intervention site care providers receive annual training and supply perspectives and knowledge elements quarterly. At the hospital level, intervention sites participate in quarterly focus groups, morbidity and mortality case reviews and quality improvement efforts, and regular motivational interviewing (MI) check-ins with site PIs.

#### Criteria for discontinuing or modifying allocated interventions {11b}

The PEGASUS pathway does not prescribe specific drug doses but provides guidelines that inform the clinical assessment and treatment for individual patients. Should a patient or their family withdraw their consent, any previously collected data is removed from analysis and, if they are a patient at an intervention site, the PEGASUS clinical pathway is no longer used at the bedside, and they receive needed, usual care.

#### Strategies to improve adherence to interventions {11c}

As a pragmatic implementation science study with quality improvement efforts, the components of the study are designed to be adaptable to support sites’ needs and to evaluate process measures to address reach (number of patients), dose (% processes implemented), and fidelity (variance from recommended processes and timely clinical pathway adoption). The Theoretical Domain Framework (TDF) guides these efforts [18].

#### Relevant concomitant care permitted or prohibited during the trial {11d}

There are no limitations or exclusions for concomitant treatment of additional injuries or diseases.

#### Provisions for post-trial care {30}

N/A. No harm anticipated; no provisions prepared.

#### Outcomes {12}

For aim 1, the main outcome is the TBI guideline adherence composite score calculation based on the BTF indicators and secondary outcomes are discharge and 3-month Glasgow Outcome Scale Extended Pediatric (GOSE-Peds) scores. Aim 2 defines and evaluates value-added TBI care processes in PICUs. Aim 3 is a real-world best practice blueprint for guideline adherence with iterative computer simulations for how relative changes in provider and organizational activities impact the magnitude, direction, and choice of operations downstream in TBI care and patient outcomes. The main unit of analysis is the clinical site.

#### Participant timeline {13}

**Fig. 2:**
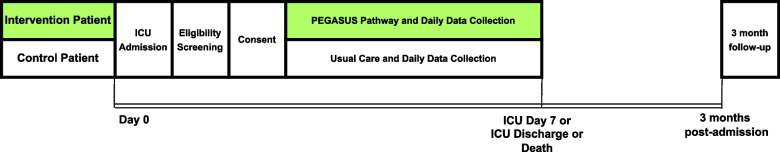
Patient timeline

**Fig. 3:**
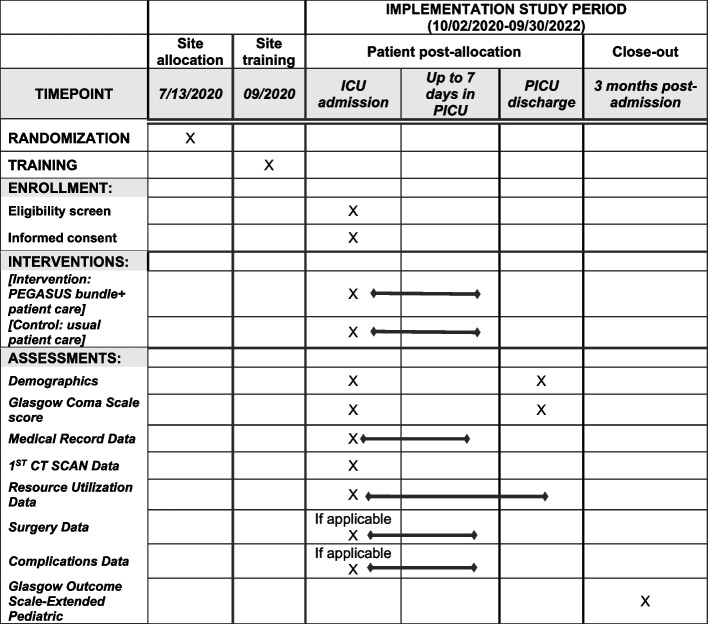
Schedule of enrollment, interventions, and assessments

PEGASUS is an in-patient pathway intervention, so site PIs and study coordinators have been trained to screen, consent, and enroll patients presenting for admission to the PICU. The intervention begins at admission and continues daily until (1) 7 days in the PICU, (2) PICU discharge, or (3) death, whichever is shortest. There is one follow-up that is completed in-person or by phone at 3 months post-admission (Figs. 2 and 3).

#### Sample size {14}

Sample sizes were derived from pre-study hospital admissions and guideline adherence estimates and assumed no change in guideline adherence at control sites. We will target recruitment of 576 patients at 16 sites during 13 months of pre-intervention data collection and 36 months of intervention implementation and data collection. We will be sufficiently powered to detect a significant difference in overall guideline adherence (main outcome) at the site level (option 3, Table 2).

#### Recruitment {15}

Ten sites were added to the original proposal to increase the potential patient recruitment population. Regular communication between hospital sites and CIIC reinforces screening and consent procedures to ensure all eligible patients are identified and consented in a timely manner.

### Assignment of interventions: allocation

#### Sequence generation {16a}

N/A. Computer-generated randomization allocated sites to either control or intervention at one time point. Blocks were stratified based on the sites’ pre-intervention baseline enrolment to balance recruitment across arms (Fig. 1). All patients admitting to a particular hospital are assigned to the same arm.

#### Concealment mechanism {16b}

N/A. No additional randomizations were required.

#### Implementation {16c}

The study biostatistician performed randomization to allocate 8 sites to the intervention and 8 sites to the control arm. The final number of patients in each arm will be dependent on eligible patients screened and enrolled by site PI and coordinators at each hospital.

### Assignment of interventions: blinding

#### Who will be blinded {17a}

Due to the nature of the intervention, participants, care providers, site PIs, and the study group are unmasked to the arm assignment of the site.

#### Procedure for unblinding if needed {17b}

N/A. Blinding not in place.

### Data collection and management

#### Plans for assessment and collection of outcomes {18a}

Both qualitative and quantitative methods are used to gain a more robust understanding of the intervention strategy. The primary data sources are hospital medical records abstracted to complete database forms and, for the intervention sites, the physical PEGASUS pathway packets based on the BTF guidelines, collected by trained PICU care providers. Site PIs and study coordinators are trained annually to perform the data abstraction and respond to data discrepancy inquiries.

#### Plans to promote participant retention and complete follow-up {18b}

Study coordinators collect 3-month follow-up GOSE-Peds for both arms using patient/family contact information at consent and confirmed at discharge. In-hospital data from participants will be included unless consent is withdrawn, even if follow-up data is unavailable.

#### Data management {19}

The purpose-built, password-protected database is maintained on a dedicated server and has logic coding and input parameters to ensure legal variable values. Qualitative data for the provider- and hospital-level intervention support include free text response in questionnaires, and transcription, notes, and summaries of focus groups and motivational interview check-ins. Data completion is assessed monthly by University of Washington (UW) and CIIC study staff.

#### Confidentiality {27}

Participant identification codes are assigned at the sites at the time of screening. Hospital site PIs and study coordinators have access to personal identifiers of their site’s enrolled patients and medical records through their dual roles as researchers and care providers. CIIC and US-based study staff have access to the full dataset through the PEGASUS database, but without protected personal information like names, birth dates, or contact information.

#### Plans for collection, laboratory evaluation, and storage of biological specimens for genetic or molecular analysis in this trial/future use {33}

N/A. No biological specimens collected.

### Statistical methods

#### Statistical methods for primary and secondary outcomes {20a}

The intervention arm (receiving the PEGASUS program) will be compared against the control (receiving usual care) for analyses. Descriptive statistics will be performed for all measures at the facility level, within treatment and control arms. Potential differences in patient (age, sex) and injury characteristics will be examined using bivariate analyses by site using χ^2^ tests (categorical) and *t*-tests (continuous). Intent-to-treat (ITT) analysis will utilize Poisson regression with robust variance estimation and clustering by site to estimate the relative risk of PEGASUS implementation on TBI guideline adherence. Sensitivity analyses to assess robustness of results will be performed.

#### Interim analyses {21b}

No interim analyses on the primary outcome (guideline adherence) are planned.

#### Methods for additional analyses (e.g., subgroup analyses) {20b}

We will perform secondary analyses including Poisson regression with robust variance estimation and clustering by site to estimate separately the relative likelihood of program implementation on these outcomes: (1) in-hospital mortality and (2) GOSE-Peds at 3 months post TBI, analyzing Glasgow Outcome Scale (GOS) score as a dichotomous measure (minor-moderate impairment vs major impairment-vegetative state and death). Multivariable Cox regression model will examine the marginal effect of PEGASUS on mortality during the 3 months post discharge, averaged over all sites. Integrating facility and provider level data, we will also test for significant mean differences in barriers and facilitators by provider, provider type (nurse vs. doctor), and site factors. We will calculate multilevel Poisson regressions to examine the relationship between the TDF constructs (measured by provider Determinants of Implementation Behavior Questionnaires (DIBQ) and each outcome (4 models at provider-level, one model at site-level). Models will include individual and site-level covariates: age, sex, injury severity (injury severity score (ISS), and head abbreviated injury score (AIS)); regional differences; and rural/urban status as appropriate.

Qualitative focus group and MI data will be coded both deductively using TDF constructs and inductively to allow for emergent themes. Themes will be integrated and compared for agreement and consistency on barriers, facilitators, and important factors associated with factors associated with TBI adherence with quantitative results.

#### Methods in analysis to handle protocol non-adherence and any statistical methods to handle missing data {20c}

We will report any differences in consent, withdrawal, and loss-to-follow-up by randomization arm. Our monthly data check process allows us to identify missing data and refer to medical records to complete data collection. The baseline data collection period had minimal missing data, so we do not have plans for imputation for missing data during implementation.

#### Plans to give access to the full protocol, participant level-data and statistical code {31c}

Access to a cleaned, deidentified dataset, and code may be requested after the study is completed and accompanying manuscripts are published. The primary aim is a study-wide analysis and will be disseminated as such. We do not expect to have sufficient data for publication at the level of individual sites. Any publications and presentations prior to the release of the primary results will not impede the integrity of those results.

### Oversight and monitoring

#### Composition of the coordinating center and trial steering committee {5d}

The study PIs are responsible for the overall conduct of the study. UW serves as coordinating center for the study direction, development and training of intervention, data management and analysis, and co-investigator activities. UW communicates internally and with CIIC at least weekly. Study PIs and CIIC meet biweekly, and the PEGASUS Argentina study team meets monthly. CIIC conducts day-to-day management of intervention delivery and implementation of activities, data entry support, training of study site staff, and direct weekly communication with study sites. All-team meetings with PIs, study team, CIIC, and site PIs and coordinators (separated by study arm) are annual.

#### Composition of the data monitoring committee, its role and reporting structure {21a}

An internal Data and Safety Monitoring Board (DSMB) adapted for pragmatic clinical trials meets with the study PIs at least biannually. The DSMB is comprised of two experienced senior investigators, Drs. Rivara and Jaffe, who are familiar with, but not part of the PEGASUS study group.

#### Adverse event reporting and harms {22}

Given that the trial examines the value of a streamlined, best-practice care pathway versus usual care, additional risks to patients are low, but patients across both arms may report adverse events related to TBIs. We have pre-intervention baseline data to assess adverse events related to patient safety or concerning trends in recruitment or follow-up data and we do not expect higher than baseline adverse events. Adverse events are reported into the database by the site PIs via a complication form for each participant enrolled into the study through discharge. Study PIs and the DSMB reviews and determines whether adverse events are anticipated for severe TBI or unanticipated. Implementation fidelity will not be monitored by the DSMB because effectiveness and implementation in real world practice is part of the study goal. DSMB reports and annual renewals are submitted the UW IRB to remain in good standing.

#### Frequency and plans for auditing trial conduct {23}

Site communication and monitoring is conducted by CIIC to educate, support, and solve problems with data collection, intervention implementation, and quality improvement, with additional input from study PIs and co-investigators as necessary. In-person site visits are conducted as feasible due to traveling restrictions.

#### Plans for communicating important protocol amendments to relevant parties (e.g., trial participants, ethical committees) {25}

Major amendments, including the study design changes described in this manuscript, were reported to IRBs, the DSMB, Clinical Trials, and our NIH National Institute of Neurological Disorders and Stroke (NINDS) program officer after being presented at a full study group meeting. We used the SPIRIT Checklist to assist with presenting this protocol [19].

#### Dissemination plans {31a}

Deidentified recruitment data is submitted regularly to NINDS Human Subjects System. At the end of data collection, we will submit data to the Federal Interagency Traumatic Brain Injury Research (FITBIR) Informatics System. In addition to the planned publication of primary findings of the trial, the study team is regularly publishing accompanying articles and analyses making use of the full extent of the data collected. The site PIs based at the participant hospitals are invited to engage with the manuscript process and sharing at their sites. Publishing plans include both PubMed-indexed English-language venues and Spanish-language journals in Argentina.

## Discussion

The stepped wedge design was responsive to local requests, the argument for equity in research, and the need to provide improvements in TBI care. In spring 2020, the study group was challenged to make decisions about the direction of the PEGASUS Argentina study design while trying to navigate many unknowns of an unprecedented situation.

### Statistical design considerations

Changing the study from the stepped wedge to a two-arm design prioritized the ability to feasibly deliver the PEGASUS intervention without delay and to assess it with sufficient power and analytical integrity. The comparison of power and detectable differences enhanced our confidence in this course of action. Delay or further interruptions as the pandemic evolved, while retaining the stepped wedge design, could have introduced confounding by the landscape of lockdowns, hospital resource strain, and other COVID-19 impacts as the sites would enter the intervention at multiple timepoints. The two-arm cluster design used the baseline recruitment data to balance the two arms at intervention, and all eight intervention sites were started on the implementation at the same time. Although sites could still be impacted by COVID-19 constraints, these impacts are more easily controlled for in analysis with the parallel control and intervention arms.

### Site and logistical considerations

During the baseline data collection period, the study group had built communication and trust with the recruiting sites. At this point, when the drawbacks to the stepped wedge design outweighed the accommodation of all sites’ eagerness to receive the intervention, site PIs and local IRBs agreed to the modifications advised due to the situation. We ensured in our planning that sites assigned to the control arm will receive PEGASUS training and initial support at the end of the study. Since our study always had remote communication components due to study group members in Rosario, Argentina, Seattle, Washington, and Washington, D.C., expanding to entirely remote training for the site PIs in South America was feasible. The technological training options alleviated some of the burden of training the eight intervention sites within a narrow time window.

### Contribution and future

This manuscript contributes to the still incomplete literature of how non-COVID-19 research studies responded to interruption and barriers on the wide scale of the pandemic. We reviewed available literature to aid in our decision-making. However, in spring 2020, many researchers were navigating widespread halts to ongoing research until aspects of study protocols could be assessed and/or adapted to reduce perceived and actual risks of COVID-19 in clinical settings [20]. There was limited information on study design changes due to wide-scale interruptions to the research landscape, much less specific to the COVID-19 pandemic. While our study group was considering options, the duration of a possible delay to our intended study design was unknown and impossible to predict. At that point, no recruiting trials had yet reported status changes due to COVID-19 to clinical trials. One year after the onset of widespread research disruption, there were nearly 1800 suspended trials due to COVID-19 [21]. Depending on the protocol, recruitment, intervention timing and delivery, and data collection methods trials were broadly affected, even if they were able to continue. One assessment of a network of trials focused on adults at-risk for cognitive decline, noted changes to recruitment plans, timeline interruptions, and remote intervention and assessments in their trials [22]. Other recent literature presented an action plan for significant trial events based on a previous trial suspension [23], and how the group was able to apply that to on-going trials during the COVID-19 pandemic [24], but these were not yet published at the time of our decision-making process.

**Fig. 4:**
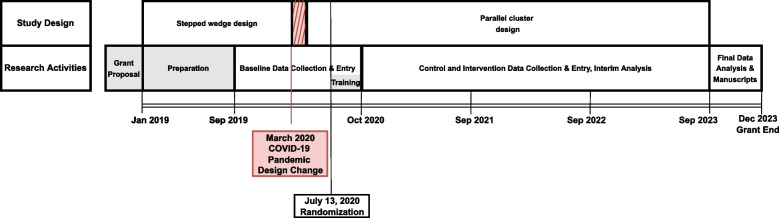
Timeline of study design changes and decision-making

The opportunity to make major design changes to mitigate the impact was reasonable due to the relatively early stage of the study (Fig. 4). Another study may not have that flexibility, limiting generalizability to studies at other stages. We continued to collect data during the transition between the study designs, so we have pre-intervention data from all sites for September 2019–September 2020. Although the PEGASUS intervention itself could not be delivered remotely, due to the focus on severe TBI in a PICU setting, our study population was still being recruited and the critical intervention components were able to embed into the essential clinical care being provided by the existing PICU team.

PEGASUS Argentina now has completed two years of the intervention with over 1 year remaining. TBI injuries typically fluctuate seasonally, as well as differ by site, so monthly recruitment is expected to vary. Over the first year (October 2020–September 2021) of intervention data collection recruitment was 93% of the baseline recruitment and because the intervention was not delayed the 3-year-long intervention recruitment period was maintained. For the most part, remote training has been able to stand-in for the original in-person training successfully. Beyond the design change, most modifications related to remote delivery of training-of-trainer and quality improvement aspects and increased reliance on communication technology in place of in-person monitoring. A secondary aim relying on additional in-person training and personnel in a subset of PICUs was delayed but will be underway soon.

The COVID-19 pandemic has had a large impact on individual health, society, economy, and public health practice, as well as on medical and public health research. A proliferation of research focused on the disease, prevention, treatments, vaccines, and other closely related issues continues to be published and informs the on-going response. However, throughout the past 2 years, research on other critical medical areas has continued. In-progress research studies faced difficult decisions to adapt design, recruitment strategies, interventions, and other components in order to continue, with limited guidance on how to make those decisions.

## Conclusions

To account for the widespread impact of the COVID-19 pandemic in South America and the constraints on research, the PEGASUS Argentina study needed to adapt designs that balanced scientific rigor with feasibility and acceptability and was able to adapt the study design to allow for training, intervention implementation, and statistical analysis plans. The experience of navigating non-COVID-19 research during the pandemic will shape the processes and decisions researchers will make about future studies to prioritize design resilience and strategies to respond to unexpected secular challenges.

## Trial status

This is protocol V.3 June 2020. Baseline (no intervention) recruitment began September 1, 2019. All intervention sites received remote training at the same time in September 2020 and transitioned to the intervention October 1, 2020. Control sites continue with usual care and data collection. Last recruitment is planned for September 30, 2023.

## Supplementary Information

**Additional file 1.** Model consent document-English translation.

## Data Availability

Only site PIs and study coordinators have access to personal identifiers of enrolled patients and their medical records at their sites in their dual roles as researchers and care providers. The study group at CIIC and UW has access to the database for the full dataset, but without the personal identifiers.

## Abbreviations

AIS: Abbreviated injury score
BTF: Brain Trauma Foundation
CIIC: Centro de Informática e Investigación Clínica
DIBQ: Determinants of Implementation Behavior Questionnaire
DSMB: Data and Safety Monitoring Board
FITBIR: Federal Interagency Traumatic Brain Injury Research
GCS: Glasgow Coma Scale
GOS: Glasgow Outcome Scale
GOSE: Peds-Glasgow Outcome Scale extended Pediatric
ICC: Intraclass correlation coefficient
IRB: Institutional review board
ISS: Injury severity score
ITT: Intent-to-treat
MI: Motivational interview
NIH: National Institutes of Health
NINDS: National Institute of Neurological Disorders and Stroke
PEGASUS: Pediatric Guideline Adherence and Outcomes
PI: Principal investigator
PICU: Pediatric intensive care unit
RCT: Randomized controlled trial
TBI: Traumatic brain injury
TDF: Theoretical Domain Framework
UW: University of Washington
VSM: Value stream map
