# Supplementary material for: The Pediatric Guideline Adherence and Outcomes (PEGASUS Argentina) program in severe traumatic brain injury: study protocol adaptations during the COVID-19 pandemic for a multisite implementation-effectiveness cluster randomized controlled trial
Source: Trials. 2022 Dec 5;23:980. doi: 10.1186/s13063-022-06938-x (PMC9720928; doi:10.1186/s13063-022-06938-x)
Supplement: Supplementary file 1 — Additional file 1. Model consent document-English translation. [file 13063_2022_6938_MOESM1_ESM.docx]

**Appendices:**

**Appendix 1.** Model consent document-English translation

**Centro de Informática e Investigación Clínica**

University of Washington

**Consent & Authorization Form**

**TITLE: Pediatric Guideline Adherence and Outcomes (PEGASUS) Project in Traumatic Brain Injury**

| **INVESTIGATORS**: | \| Monica Vavilala, MD- University of Washington  Michael Bell, MD- Children’s National Hospital \| \| \| --- \| --- \| \| Gustavo Petroni, MD, MCR -CIIC  549-341-514-7543 \| \| \| Silvia Lujan, MD  549-341-560-9239  Dr. XXX, Site PI  Hospital XXXX  City/ Country  Telephone \|  \| \| |
| --- | --- | --- | --- | --- | --- | --- | --- | --- |

**SPONSOR:** National Institute of Neurologic Disorders and Stroke

**Researcher’s statement:**

We are asking you to allow your child to be in a research study. The purpose of this consent form is to give you the information you will need to help you decide whether to allow your child to be in the study or not. Please read the form carefully. You may ask questions about the purpose of the research, what we will ask you to do, the possible risks and benefits, your child’s rights as a volunteer, and anything else about the research or this form that is not clear. When we have answered all your questions, you can decide if you want your child to be in the study or not. This process is called “informed consent.” We will give you a copy of this form for your records.

**PURPOSE:**

***Brain injury can happen when a person hits their head from a fall, a car accident, violence, or other kinds of trauma. Children are especially vulnerable to having a brain injury. The purpose of this five-year study is to examine the effect of a hospital program called PEGASUS, which aims to increase use of evidence based pediatric traumatic brain injury guidelines, and also aims to improve patient outcomes.***

We are conducting this study in 16 hospitals in Argentina, Chile, and Paraguay. We will be enrolling new participants into the study for three years. We expect to enter a total of about 540 participants into this study. This large number will allow us greater understand the effects of PEGASUS program on adherence to evidence based pediatric traumatic brain injury guidelines and discharge outcomes. Of the 16 participating sites, eight sites will receive the intervention (PEGASUS program) and eight sites will be control sites and provide data on the care they receive. In Year 5, control sites will be given the program if the analysis in year 4 shows a benefit of the program.

## ***Your child has been invited to participate in this study because he/she has a brain injury and is receiving care at one of the participating hospitals which is an intervention site.***

**PROCEDURES**:

If you choose for your child to take part in this study, your clinical care team will receive a checklist of clinical care tasks outlined in a list of Traumatic Brain Injury guidelines to follow daily during hospitalization. We will collect clinical data (like information from your child’s medical record, including notes, lab results, and how they are doing after discharge). We will ask you a few questions to assess how your child was before the injury. We will ask for your contact information so we can contact you at 3 months after discharge to ask you some follow up questions over the phone to see how your child is doing.

Your de-identified data will be shared with the Investigators listed on this form. The information about your person will be confidential through the process used to keep the personal identity (e.g. name, address, identification number, or any other kind of identifying information) from being linked to a particular person. A description of this clinical trial will be available on http://www.clinicaltrials.gov, as required by U.S. Law. This web site will not include information that can identify you. At most, the web site will include a summary of the results. You can search this web site at any time. You may refuse to answer any question at any time. If you have any questions regarding this study now or in the future, contact Monica Vavilala, MD (+1 206 731 8386) , Gustavo Petroni, MD (549-341-514-7543) or Local PI XXX , (Phone XXX).

**RISKS AND DISCOMFORTS**:

**Risk of Loss of Confidentiality.** Although we have made every effort to protect your identity, there is a minimal risk of loss of confidentiality. During the time that your child will be participating in this study, his/her medical records will be associated with his/her name. The actual records that we will use for the study will have only a participant number. Those records will be stored in locked cabinets and protected computer files, and only the study personnel can see them. In a separate location, there will be a list that associates the participant’s name with the record number. We will maintain the list linking participant’s names with record numbers for 10 years, after which it will be destroyed.

At the end of the study, we will analyze the information from all of the participants in the study. We will make conclusions and report those conclusions. We will not use any personal information that identifies any participant in those analyses, conclusions, or reports.

**BENEFITS**:

You may not benefit from being in this study. If the intervention program is shown to improve outcomes in children, it can be used in the future to improve outcomes for other children. This knowledge will be important in all parts of the world.

**ALTERNATIVES**:

You may choose for your child not to be in this study. If you decide your child should not participate in this study, he/she will receive the standard care provided by this hospital.

**CONFIDENTIALITY AND PRIVACY OF YOUR PROTECTED HEALTH INFORMATION:**

We will not use your name or identity for publication or publicity purposes. Research records may be reviewed and/or copied by the Institutional Review Board or the sponsor to make sure the study is being done safely. If a review of this study takes place, your records may be examined. The reviewers will protect your privacy. The study records will not be used to put you at legal risk of harm.

We will retain much of your child’s protected health information (demographics, injury information, treatment information, physiologic data throughout treatment and in-hospital rehabilitation information) .The information about your child will be kept confidential. Your child’s file will have a number, and the number will be linked to your name on a list that is kept separate, in a locked file cabinet. We will keep this list for 10 years. Only our research team will have access to the list that links the patient with the patient’s files. All information you provide will be confidential. (U.S. Law No. 25,326 on Protection of Personal Data).

You have the right to revoke this authorization and can withdraw your permission for us to use your child’s information for this research by sending a written request to the Principal Investigator listed on page one of the research consent form. If you do send a letter to the Principal Investigator, the use and disclosure of your child’s protected health information will stop as of the date she receives your request. However, the Principal Investigator is allowed to use and disclose information collected before the date of the letter or collected in good faith before your letter arrives.

The information about your child that is used or disclosed in this study may be re-disclosed and no longer protected under U.S. federal law.

**COSTS**:

You will not receive any financial reimbursement for participating in the study. Participation in the study won’t add any additional cost to your child’s hospitalization.

**LIABILITY**:

If you think you have an injury or illness related to this study, contact the staff of this investigation immediately. The staff in charge of the study will provide the necessary treatment or refer you to whom it may concern.

**PARTICIPATION**:

If you have any questions regarding your rights as a research subject, you may contact Ethics Committee______ at ______-_________

You do not have to join this or any research study. If you do join, and later change your mind, you may quit at any time. If you refuse to join or withdraw early from the study, there will be no penalty or loss of any benefits to which you are otherwise entitled.

Your health care provider may be one of the investigator*s* of this research study, and as an investigator is interested in both your child’s clinical welfare and in the conduct of this study. .

We will give you a copy of this form.

**SIGNATURES:**

Your signature below indicates that you have read this entire form and that you agree to be in this study.

____________________________________

Child’s Full Name

______________________________________________________________________

Signature of Parent or Legal Guardian Date

___________________________________ __________________________________

Print Name Relationship to Child

_____________________________________________________________________

Signature of Investigator Obtaining Consent Date

___________________________________

Print Name
